# LINPS: a database for cancer-cell-specific perturbations of biological networks

**DOI:** 10.1093/database/baab048

**Published:** 2021-08-20

**Authors:** Mahmoud Ahmed, Deok Ryong Kim

**Affiliations:** Department of Biochemistry and Convergence Medical Science, Institute of Health Sciences, Gyeongsang National University College of Medicine, 816 Beon-gil 15, Jinju-daero, Jinju 52727, South Korea; Department of Biochemistry and Convergence Medical Science, Institute of Health Sciences, Gyeongsang National University College of Medicine, 816 Beon-gil 15, Jinju-daero, Jinju 52727, South Korea

## Abstract

Screening for potential cancer therapies using existing large datasets of drug perturbations requires expertise and resources not available to all. This is often a barrier for lab scientists to tap into these valuable resources. To address these issues, one can take advantage of prior knowledge especially those coded in standard formats such as causal biological networks (CBN). Large datasets can be converted into appropriate structures, analyzed once and the results made freely available in easy-to-use formats. We used the Library of Integrated Cellular Signatures to model the cell-specific effect of hundreds of drug treatments on gene expression. These signatures were then used to predict the effect of the treatments on several CBN using the network perturbation amplitudes analysis. We packaged the pre-computed scores in a database with an interactive web interface. The intuitive user-friendly interface can be used to query the database for drug perturbations and quantify their effect on multiple key biological functions in cancer cell lines. In addition to describing the process of building the database and the interface, we provide a realistic use case to explain how to use and interpret the results. To sum, we pre-computed cancer-cell-specific perturbation amplitudes of several biological networks and made the output available in a database with an interactive web interface.

Database URL https://mahshaaban.shinyapps.io/LINPSAPP/

## Background

Biological knowledge accumulates in the form of literature, ontologies, databases and datasets. Assembling knowledge in computable and programmable formats increases its utility as they make it easy to communicate (1, 5). Large-scale studies generate data that can be used to explore and develop novel hypotheses. Efforts were also made to curate and homogenize smaller datasets for the same purpose. Integrative analyses often produce more reliable insights.

Reverse causal reasoning takes advantage of the known biological concepts and existing datasets (3). In this approach, high-throughput experimental data are used to generate gene expression signatures which are integrated into biological networks to infer causality (9). Applying these methods is laborious and resource-intensive. This article describes a database and web interface of pre-computed cancer-cell-specific perturbations scores of hundreds of drugs. Those can be used to screen for effective cancer therapies and formulate hypotheses on their mechanisms of action.

## Data and Methods

### Perturbation gene expression signatures

The Library of Integrated Cellular Signatures (LINCS) is a library of gene expression profiles of multiple cells under different types of perturbations, including compounds, gene overexpression, knockdown or knockouts (7). Multiple cell lines were profiled for gene expression using the L1000 technology, which only measures the expression of 1000 genes and imputes the expression of the rest from these measurements. We only included a subset of perturbations (*n* = 19 500) of drugs with known mechanisms of action and used these information to classify the drugs into groups (*n* = 550) (Table 1). The data were obtained using the Slinky R package (8).

**Table 1.** Description of the data sources.

	Cells			Perturbations			Networks		
Desc.	Gene expression profiles of cell lines from different cancer tissues and metadata about the cell phenotypes.			Control vs. treatment gene expression profiles of cancer cell lines and metadata about the treatments.			Two-layer CBN of cellular processes.		
		N	Example		(N)	Example		(N)	Example
Content	Tissue	12	Breast/Skin	MOA	530	NSAID/Anti-ER	Family	5	IPN/CPR
	Cell	70	MCF7/A375	Drug	1938	Asprin/Tamoxifen	Model	8	Apoptosis
Source	LINCS (7)						CBN	(1)	

### Differential expression of drug treatments

**Figure 1.**
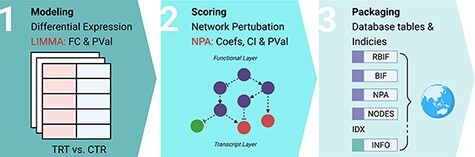
Description of the workflow. The workflow to build the database consists of three main steps. First, modeling the effect of the drug treatments on individual genes in every cell line. Differential expression analysis was applied using Limma to compare the gene expression between the treatment and control conditions. Second, scoring the fold change and *P*-values from the differential expression to calculate the network perturbation amplitudes (NPA) of the drugs on each network (NPA) and each node (nodes contributions). The sum of the NPA for a group of networks is known as the biological impact factor (BIF). Finally, the BIF, NPA and nodes contributions were packaged in a database file and metadata of the cell lines, drugs and networks.

To determine the effect of drug treatments on gene expression, we split the expression profiles by the mechanism of action and cell lines. In each group, treated samples were compared to samples from the same cell line treated with dimethylsulfoxide as control. We used Limma to apply the differential expression and calculate the fold change and *P*-values for each gene (Figure 1) (12). Correlation analysis was conducted using the fold change to generate similarity profiles of the treatments based on the gene expression changes they produce in each cell.

### Causal biological networks

Causal biological networks (CBN) are set of networks on cell fate, stress, proliferation and others compiled from the scientific literature (1). The networks are encoded and compiled in the biological expression language (BEL) (5). The current version of the database contains eight different networks belonging to five different families (Table 1). Those were obtained and preprocessed using the NPAModels package.

### Scoring the networks with expression signature

#### Motivation

Here, we assume that an entity’s function in a biological pathway (e.g. the activity of a given transcription factor) is reflected in the expression of the genes downstream. We can construct a network of two layers to describe this pathway. First, the functional layer would encode the causal relations between the entities of interest. This can be coded manually or extracted from the literature. The second is a transcription layer with all the nodes downstream from each node in the backbone. NPA models the changes of perturbation in the nodes of a biological network as the changes in expression of their known downstream nodes (Figure 1) (9). This analysis was applied using an R package with the same name (10).

#### Model (reproduced from [Martin et al., 2019])

Expression data are used to compute scores for the network nodes based on constraint optimization. This problem is solved analytically using matrix multiplication. When the transcription data reflect the perturbation of the functional layer, all differential values should be close to each other (smooth) and equal to the observed fold change *β* in the transcript layer (*V*_0_). Differential values are calculated by solving:

$$\begin{equation*} min_{f\in l^2 (V) \sum_{x\to y}} (f(x)-\sigma(x\to y).f(y))^2\vspace*{4pt} \end{equation*}$$

where $\sigma(x \to y)$ denotes the sign of the edge *x* → *y* in the network such that

$$\begin{equation*} f\,\Big|_{V_0} = \beta \end{equation*}$$

The NPA score is computed by summing the results over the edges of the functional layer

$$\begin{equation*} NPA = \frac{1}{|E|} \sum_{e\ in\ E} (f(e_0) + \sigma(e)f(e_1))^2 \end{equation*}$$

where *E* is the set of its edges and $|E|$ is its size, *f* is the solution of the constrained problem, and *e*_0_ and *e*_1_ denote the start and the end of the edge *e*.

#### Interpretation

The perturbation amplitude of a network (NPA) is a positive number that describes the degree of treatment-induced perturbation or activity of the network. Confidence interval (CI) is calculated around the score based on the variance in the differential expression values. CI should not contain zero for significantly perturbed networks. Leading nodes are the functional layer nodes with differential values that contribute the most to the NPA scores (>80%). Finally, when applying the analysis to multiple networks, the BIF is the weighted sum (0 to 1) of the significantly perturbed network scores.

#### Accompanying statistics

Three statistics accompany each score. First, a CI based on the biological variability propagated from the uncertainty of the differential gene expression values. A CI that does not contain zero is considered significant. The second and third are generated by reshuffling the edges in the functional and the transcriptional layers to create null distributions. NPA values above the 95% quantile of the null distribution generated from each layer are considered significant.

## Implementation

### Database description

The differential expression output and the network perturbation analysis were stored as tables in an SQLite database file. The database was built using the DBI and dbplyr R packages (14, 15). These tables are rbif, bif, npa and nodes. Each table is indexed by the tissue and cell line, perturbation type and name, and network family and model. The coefficients from the BIF and the NPA analyses are stored in the corresponding tables with the related CIs. In addition, two metadata tables were added to keep track of the data and analysis output. perturbations contain information on the cell lines and the drugs used as treatment. models contain information of the CBN and the graph object.

### Web interface description

**Figure 2.**
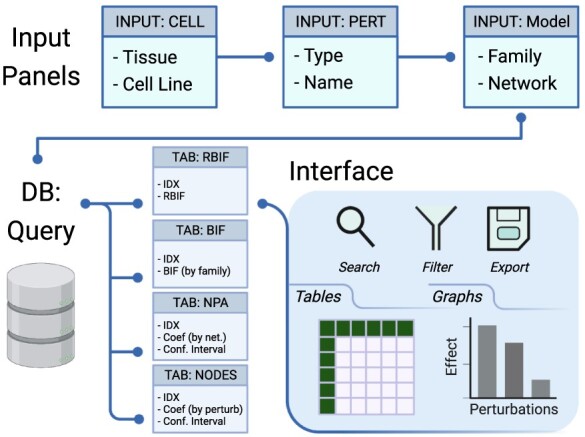
Description of the database and the interface. The interface includes input panels to query the database, and the output is sent back to the interface. The query is constructed by selecting the cell lines from one or more tissues, the drug perturbations grouped by mechanism of action, and the network models of interest. The output of the database query is three main tables: the BIF, NPA and code contributions (NODES). The output is returned in tabular and graphical formats that can be searched, filtered or exported.

The web interface to the database file was designed using Shiny (4). The application is divided into two parts for the inputs and the outputs (Figure 2). The input panels are used to specify the Tissue/Cell Line, Network Family/Model, Drug type (mechanism of action) and Name of interest. A query is interactively generated on the back end and sent to the database. The return is capture in tabular and graphic formats in the output tabs (Box 1). The first two tabs (RBIF and BIF) show the relative and the absolute cell-specific impact of a group of drugs in the selective network. NPA and NODES show the impact of the drugs on a network and individual nodes in the network, respectively. GRAPH can be used to explore further the structure of the network and the effect of individual drug perturbations. SIMILARITY between the perturbations was based on the fold change in response to drug treatments in a given cell line.

Box 1. Glossary

Input panels

- Cell
  - Tissue: the tissue name of the cell lines (e.g. Skin, Lung, Kidney, Liver, etc.)
  - Cell line: the official name of the cell line (e.g. A375, A54, HA1E, HEPG2, etc.)
- Network
  - Family: networks are grouped into categories such as Cell Proliferation (CPR), Inflammatory Process (IPN), etc.
  - Model: the name of the network (e.g. Cell Cycle, Epithelial Innate Immune Activation, etc.)
- Perturbation
  - Type: the type of perturbations or mechanisms of action (e.g. Abelson kinase inhibitor, 5’ adenosine monophosphate-activated protein kinase activator, etc.)
  - Name: the name of the particular drug (e.g. Imatinin, bosutinib, polypyrimidine tract-binding protein 1, etc.)

Output tabs

- RBIF: Relative Biological Impact Factor
- BIF: Biological Impact Factor
- NPA: Network Perturbation Amplitudes
- NODES: the nodes that constitutes the network model
- GRAPH: a graph representation of the network model
- SIMILARITY: a similarity measure between perturbations based on cell-specific gene expression changes
- DOWNLOAD: download the analysis output or the full database

Functions for searching, filtering and exporting the output are built into the interface. Tables and figures were built as interactive widgets using DT, Plotly and visNetwork to refine and export the output in a publication-ready format (2, 13, 16). In addition, a DOWNLOAD tab was added to enable downloading the individual tables or the full database in text format.

The database is designed and optimized for search. Users can screen for a large numbers of drugs, models or cell lines and evaluate their significance using the relative biological impact factor (RBIF) output tabs. Next, the other output tabs can be used to refine the selection and produce publication-ready figures and tables for the items of interest. In addition, the interactive nature of the graphs and the tables allows for remaking the output and refining them to fit multiple use cases. Finally, the output, as well as the full database, can be exported to be explored or analyzed elsewhere.

### Source code and Availability

The analysis, scoring and packaging was conducted mainly in R and using Bioconductor packages (11, 6). The software environment was packaged into a Docker image and made available at https://hub.docker.com/r/bcmslab/linps. The source code to build this image, the database and the web application is open source (GPL-3) and is available at https://github.com/BCMSLab/LINPS and https://github.com/BCMSLab/LINPSAPP. The interface can be accessed locally through a Shiny Server docker image (https://hub.docker.com/r/bcmslab/linpsapp) or online (at https://bcmslab.shinyapps.io/LINPSAPP/).

## Use case

### Motivation: tyrosine kinase inhibitors arrest the cell cycle of skin cancer cells

Tyrosine kinase inhibitors target the Abelson (Abl)-Bcr kinase, which is a chimeric oncogene of the *Abl gene* at chromosome 9, and the *Bcr gene* at chromosome 22 common in the development of chronic myelogenous leukemia. The first of those drugs to be developed was Imatinib. Other drugs followed to overcome the Imatinib resistance and limitations. Secondary and complementary mechanisms of action were reported to benefit patients with other conditions. In this use case, we focused on the Abl tyrosine kinase inhibitors and their potential mechanism of action in cancer. We studied the effect of the treatment on biological pathways and in cellular contexts other than their previously identified targets. Our interface was able at once to compare and contrast multiple drugs and to show in detail the possible pathway and cellular context of the significant effects.

### Choosing cells, networks and drugs inputs

**Figure 3.**
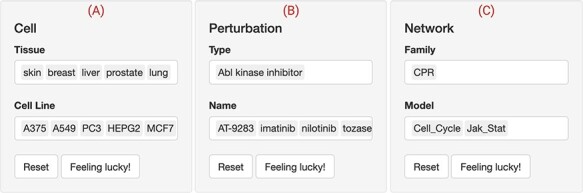
The web interface input panel. The interface contains a main input panel which is divided into three parts. (A) Cell is to choose the tissue and cellular context where the drug perturbations would be shown. (B) Perturbation allows for searching and selecting the type (mechanism of action) and name of the drugs of interest. (C) Network lists the available biological networks to be scored classified into multiple families. Multiple choices, resting and selecting a few entries at random are allowed. In this use case, five ‘Abl kinase inhibitors’ (‘AT-9283, imatinib, nilotinib, tozasertib and ZM-306 416’) were selected to check whether they perturb the cell proliferation (‘CPR’) through the ‘cell cycle’ and ‘Jak-Stat’ biological networks in five (‘A375, A549, PC3, HEPG2 and MCF7’) cancer cell lines.

We first used the web interface to filter for the cell lines that are responsive to Abl inhibitors through the RBIF output (not shown). RBIF showed the relative effect of every perturbation on the included networks. We compared and contrasted several permutations of the drugs, cell lines and network inputs. The output suggested a potential effect of these inhibitors on the CPR networks in five cell lines. The skin cancer cell line ‘A375’ and the CPR ‘cell cycle’ network were further explored (Figure 3).

### Interpreting impact factors, perturbation amplitudes and node contributions

**Figure 4.**
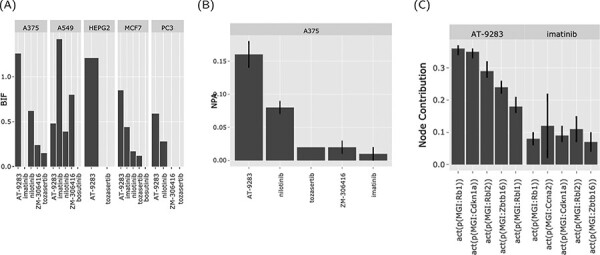
The web interface graphical output. The output of the ‘Abl kinase inhibitors’ on the CPR networks in five cancer cell lines (as selected in Figure 3). (A) BIF of every drug on the CPR networks stratified by the cell line. (B) NPA of every drug on the ‘cell cycle’ biological network in A375. (C) The relative contribution of each node (top five) in the cell cycle network in response to the drug perturbations in A375.

The BIF shows the impact of the drugs that share a mechanism of action on a family of biological networks (cell cycle and Jak stat) in different cell lines. In this case, two drugs in particular (nilotinib and AT-9283), a second-generation Ab inhibitor and a drug that targets Abl as well other kinases, respectively (Figure 4A). The two drugs perturb the cell cycle network (AT-9283, NPA > 0.15 ± 0.01 and nilotinib, NPA > 0.05 ± 0.005) (Figure 4B). This indicates that a sizable fraction of the nodes in the cell cycle network was perturbed (activated or inhibited) in response to treatment with these drugs. The effect was strongest in the A375 skin cancer cell line but was also observed in other cell lines. To further investigate the possible pathways for this significant effect, we looked at the nodes’ contributions. Notably, the retinoblastoma 1 (RB1) and like 2 (RBL2) protein activity were the highest contributors (Figure 4C).

### Analyzing the perturbed network

**Figure 5.**
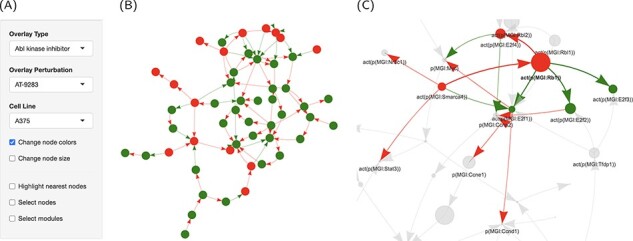
Graph representation of the network perturbations. (A) Input panel to search and select from the main inputs for individual perturbations to visualize on the network graph. (B) The ‘cell cycle’ network shown as a graph overlaid with the effect of ‘AT-9283’ in the ‘A375’ cell line. (C) The same with the highlighted cluster. Nodes are colored by the direction of regulation in response to the drug treatment (red, up, and green, down-regulated). Edges are colored by regulation direction between the nodes (red, increase; and green, decrease).

High contributions indicate a significant role for these proteins in perturbing the cell cycle. To get an overview of the treatment effect of this pathway, we used the graphic representation capabilities in the interface to specifically overlay the perturbations caused by AT-9283 on the cell cycle network (Figure 5A). It turned out that most of the network nodes were turned off in response to the drug treatment, possibly through the action on a handful of key proteins (Figure 5B). The activation of RB1 resulted in the repression of the activity of three of the E2F Transcription Factor family proteins, which themselves were downregulated by AT-9283 (Figure 5C). The graph interface makes viewing a large number of interactions possible. In addition, overlying the computed scores in the form of node size and color increases the visibility of significant trends. A clustering algorithm (fast greedy) was also implemented to partition the network into connected modules.

RB1 is a negative regulator of the cell cycle that directly binds to the E2F transcription factor and represses its transcriptional activity. Phosphorylation/activation of RB1 by some cyclin-dependent kinases releases E2F from the RB1/E2F complex and consequently promotes the transcriptional activation of target genes necessary for cell cycle progression. It is possible also that RB1 works indirectly through RBL2 and MYC, which was induced by the same drug treatment, to produce a similar effect.

### Remarks on the interpretation

- The causal biological network encodes the entities in the network (nodes) and the directed relation between them (edges). Those were either identified manually or extracted from the literature and did not change with the treatment itself, but rather
- The NPA method uses the gene expression of all the nodes connected to a specific entity to infer its function (activation or inhibition).
- Considering the two pieces of information, we could determine that a particular node is activated, inhibited or unchanged to produce a specific function that is encoded a priory.
- Taken together, we could conclude that the cancer cell line’s cell cycle of A375 cells might be arrested in response to AT-9283 treatment through the negative regulation of RB1 on the E2F transcription factor. This effect was present to a lesser extent with other Abl inhibitors and in other cancer cell lines.

## Limitations and future directions

This analysis is only valid insofar as the underlying data (gene expression and causal links) reflects the true biology of the conditions and biological functions. The expression of most genes was only inferred from the measurement of landmark genes and the causal networks were manually compiled from the literature; therefore, it is possible to miss known or recently identified interactions. In both cases, due to methodological limitations, the underlying biology is never completely represented in the form of graphs or expression profiles.

The database contains pre-computed scores of existing networks and perturbations. It is not possible to create or customize the scores to other networks or datasets through the web interface. Due to resource limitations, the relative impact of the drugs was restricted to other drugs in the same group (mechanism of action) and not to all other drugs. Similarly, we did not include the dose or period of treatment in the differential expression model. Finally, we computed the scores for cell lines separately due to the large size of the datasets.

In future releases, we plan to expand the database to include more perturbations and biological network models. LINCS datasets comprise other drug and ligand treatments as well as genetic perturbations (knockdowns, knockouts and overexpression). Biological networks such as those of autophagy, DNA damage and repair will also be scored and added to the database. Furthermore, in the current version of the database, we did not consider the dose and treatment period. It would be useful to determine the precise dose and duration of treatment that produce the desired effect. Finally, more network models can be added either from existing CBN or by converting protein–protein interaction databases into the BEL.
